# Environmental Value and Pro-environmental Behavior Among Young Adults: The Mediating Role of Risk Perception and Moral Anger

**DOI:** 10.3389/fpsyg.2022.771421

**Published:** 2022-02-09

**Authors:** Xin Li, Zhenhui Liu, Tena Wuyun

**Affiliations:** ^1^School of Psychology, Inner Mongolia Normal University, Hohhot, China; ^2^Department of Psychology, Honghe University, Mengzi, China

**Keywords:** environmental value, risk perception, moral anger, pro-environmental behavior, structural equation modeling, Chain mediating effect

## Abstract

This study aims to identify the relationship between students’ environmental value (EV) and pro-environmental behavior (PEB) within a values-belief-norm framework. To conduct an empirical study, we used a sample of 558 online surveys and adopted the partial least squares path modeling method to test the relationships between variables in the conceptual model. The results indicate that EV positively predicted PEB among young adults. In addition, we highlight that risk perception (RP) and moral anger (MA) play critical chain mediating roles in the relationship between EV and PEB. This study has meaningful implications for practitioners seeking to encourage the public’s ecofriendly behavior by suggesting ways to encourage RP and stimulate individuals’ moral emotions about the environment.

## Introduction

Environmental issues are related to the sustainable development of human society. Environmental risk has ranked first on the World Risk List for 5 years in a row, just as the latest Global Risk Report, which was released by the World Economic Forum in 2021, pointed out that in the next decade, extreme weather, biodiversity loss, natural resource crises, and climate action failure will continue to dominate. Clearly, these environmental problems primarily stem from human activities (Dong et al., 2017), such as driving cars, energy use, diet, household waste and other behaviors. Such a severe ecological crisis has not only aroused widespread concern among the public but also prompted researchers to probe deeply into mechanisms of individual pro-environmental behavior (PEB) to provide practical strategies for improving the condition of the environment (Wang and Wu, 2015). College students are the main source of contemporary and future environmental protection and an important group for practicing environmental protection. Recent studies have found that young people are more concerned about the environment than older generations (Royne et al., 2011), and young people are considered to be the promoters of new environmental movements, such as green consumer activities (Bentley et al., 2004). Therefore, we examine Chinese college students’ PEB to inspire their understanding of the importance of environmental protection and help them form stable values, stimulate their moral awareness and sense of social responsibility, and engage in more environmentally friendly behaviors.

Pro-environmental behavior refers to the behavior that individuals need to be conscious, voluntary, and active in avoiding harming the environment (Steg and Vlek, 2009; Liu and Wu, 2013). PEB covers many aspects of environmental protection and can be carried out through direct and indirect means to mitigate environmental damage; it promotes harmonious coexistence between humans and nature. Studies have confirmed that PEB is influenced by cognitive factors, including environmental value (EV; Mustonen et al., 2016), environmental identity (Clayton, 2012), environmental concern (Coelho et al., 2017), and awareness of consequences (Chen and Tung, 2014). Among these factors, an individual’s EV is the most stable factor and is considered the most potent predictor of PEB (Han et al., 2018; Riper et al., 2020). The basic theory of values (Stern and Dietz, 1994) also holds that PEB is a form of prosocial behavior caused by internal values. It has been found that biospheric values (i.e., concern about the ecosystem’s interests) can positively predict pro-environmental intention and behavior (Geiger and Keller, 2018). However, egoistic values (concern only for one’s own interests) are negatively related to environmental protection behavior (Hurst et al., 2013). Individuals with low EV are indifferent to the ecosystem’s interests, so they are less likely to consciously care about the environment or adopt behaviors that are beneficial to the environment.

Subsequent studies have uncovered that although values are essential to behavior, values do not necessarily directly translate into behavior, and there can be a gap between values and behavior (Yousefpour et al., 2019). For example, individuals can strongly cherish nature and the environment but not think of themselves as people who support environmental protection (Juvan and Dolnicar, 2014). According to the values-belief-norm theory, other factors affect PEB, such as beliefs (Ünal et al., 2018), personal norms (Antonetti and Maklan, 2014), perceptual control (Du et al., 2020) and other cognitive factors. The studies show that only individuals with high EV, certain environmental beliefs, moral norms, and behavioral and perceptual control can promote PEB. Therefore, other intermediary mechanisms may be involved in the relationship between EV and PEB, as mentioned in Ünal et al. (2018).

In early research on the relationship between EV and PEB, researchers paid considerable attention to the role of cognitive factors. However, in many situations, individuals do not act in a completely rational way. Individuals do not fully understand the relationship between their environmental decisions and environmental outcomes and may be influenced by morality or emotion (Onwezen, 2015).

A recent study (Yu and Tian, 2017) found that the role of emotional factors such as guilt (Adams et al., 2020), compassion (Geiger and Keller, 2018), and moral anger (MA) should not be ignored (Reese and Jacob, 2015). Some studies have confirmed that cognitive and emotional factors together play a role in EV and PEB (Lerner et al., 2015; Riper et al., 2020). There is evidence that individuals with high EV have a stronger sense of risk and are more likely to perceive the threat posed by the environment, which makes people aware of the consequences of their own harmfulness and motivates them to engage in more PEB (Schmitt et al., 2017, 2019). Zhang (2013) also found that individuals with higher EV perceive environmental risks significantly more than other people and pay more attention to environmental issues, making them less likely to take risks in environmental decision-making. Therefore, we believe that risk perception (RP) plays a mediating role in the relationship between EV and PEB. In addition, moral emotion also has an influence on PEB (Onwezen et al., 2013; Han et al., 2018). Some studies have shown that in the face of environmental damage, individuals’ strong belief in environmental justice will stimulate greater MA and a sense of responsibility and will prompt more pro-environmental intentions (Reese and Jacob, 2015). Atran and Ginges (2012) believed that when some protective values are threatened, an individual will produce a series of negative emotions (such as anger and disgust) to fight (Sheikh et al., 2012) and then trigger the corresponding behavioral response. Therefore, we have reason to believe that MA may also partly explain the relationship between EV and PEB. Therefore, RP and MA may play multiple mediating roles in the relationship between EV and PEB. Our research mainly reveals the intermediary mechanism between EV and PEB.

Because of the focus of these studies, the overall goal of this study is to establish a conceptual framework including cognitive and emotional processes to clearly understand the mechanism of college students’ PEB. We studied 568 college students and explored the possible relationship between RP and MA according to the theoretical model and hypothesis proposed in previous studies, and we investigated the mediating role of RP and MA between EV and PEB. Exploring the intermediary mechanism between EV and college students’ PEB can provide guidance for the exploration of relevant theoretical mechanisms of environmental psychology and can provide new ideas for the stimulation of young people’s PEB. Such work also provides methods and guidance for the treatment of environmental pollution in China.

## Literature Review and Hypothesis Development

### The Relationship Between Environmental Value and Pro-environmental Behavior

Values refer to a series of important life goals or standards that play a guiding role in people’s lives (Schwartz, 1992). EV is one such value, and it is the most stable and lasting guiding principle of individual environmental behavior. According to de Groot and Steg (2008), EV is an ideal condition for dealing with the relationship between human beings and the ecological environment. The term refers to the degree of value individuals ascribe to issues related to the biosphere and the environment.

Environmental value has long been regarded as the driving factor of moral norms and the cognitive factor of decision-making regarding environmental protection. It can guide behavior by promoting the evaluation of situations and can lead to goal setting (Han, 2015; Fornara et al., 2016). Many studies have confirmed that EV is an important predictor of PEB (Pradhananga et al., 2017; Ünal et al., 2018). Individuals or groups with positive EV will show more PEBs (de Groot and Steg, 2010). Compared to environmental knowledge, environmental awareness, environmental concern, and other variables, EV has more positive predictive power and can more effectively predict support for environmental protection, including pro-environmental policies (Steg et al., 2011; Hurst et al., 2013). Individuals who attach importance to EV often regard environmental protection as an essential pursuit. A person who cares greatly about the environment engages in more environmentally friendly behaviors to protect the environment and carries out more PEBs than others. However, the current research shows that EV cannot fully explain PEB because behavior can also be influenced by other intermediary variables (e.g., cognitive and emotional factors). Thus, it is necessary to fully investigate the relationship between EV and PEB; meanwhile, it is important to identify the contributions of cognitive factors such as RP and emotional factors such as MA to this association.

Thus, we propose the following hypothesis:

H1. **Individuals’** EV **is related to** PEB.

### The Mediating Role of Risk Perception in the Relationship Between Environmental Value and Pro-environmental Behavior

Risk perception, a more authoritative term, was first proposed by Slovic (1999) and refers to people’s empirical judgments of and attitudes toward various risks. The concept explains how the public perceives social threats (Wachinger et al., 2013; Slovic, 2016). Many studies have confirmed that RP is a multidimensional cognitive structure. This study refers to the studies of Yu and Xie (2006) and Zhang (2013); environmental RP refers to an individual’s perception of the severity, possibility, and persistence of risk, which can reflect an individual’s psychological representation of environmental hazards.

Previous studies have found that EV can predict an individual’s willingness to drive an ecologically friendly car because it is highly related to perceptive consequences of driving cars, such as exhaust emissions (Ünal et al., 2018). RP plays a mediating role in the relationship between EV and ecofriendly driving. Individuals who attach great importance to EV and consider themselves a part of nature will care about environmental issues, and they are more sensitive to the threats of the environment (Tortosa-Edo et al., 2014). Some studies have shown a strong correlation between EV and RP (Liu et al., 2014; Ojala and Lidskog, 2017). According to the social amplification of the risk framework, EV is an essential factor in perceiving the risks of global warming (Ohe and Ikeda, 2005). When an individual feels threatened by risks to his important values, his risk awareness will increase (Mumpower et al., 2016). If confrontation with environmental risk threatens the important values that people hold, people’s perception of risk will decline. For example, individuals who focus on hedonistic values have less concern about environmental problems (Islam et al., 2013).

Recent studies have shown that RP is positively related to individuals’ PEB (Pahl et al., 2005; Schmitt et al., 2019). For example, there is a significant positive correlation between the public’s RP of marine microplastic pollution and pro-environmental intention (Jeong et al., 2021). There is a positive correlation between the perception of flood risk and the willingness of individuals to take private mitigation measures (Terpstra et al., 2009; Bubeck et al., 2012). According to the theory of protection motivation, people will adopt defensive action to reduce their risk when faced with a risk event. For example, stronger climate RPs may foreshadow more energy-saving behaviors (Linden, 2015; Lacroix and Gifford, 2017). Clearly, when we have stronger RPs of the environment, we will engage in corresponding PEB to reduce our environmental RP. These findings suggest that RP plays a mediating role between EV and PEB.

H2. **Environmental value is related to** RP.

H3. **Risk perception is positively related to** PEB.

### The Mediating Role of Moral Anger in the Relationship Between Environmental Value and Pro-environmental Behavior

Moral emotion refers to an emotional experience whereby an individual evaluates his or her behavior and thoughts or those of others according to specific moral standards (Haidt, 2003). Emotions aroused by assessing one’s behavior and beliefs are called self-conscious emotions and include pride, guilt, shame, and empathy (Onwezen, 2014). Emotions evoked by evaluating the actions and thoughts of others are called emotions related to others, and include anger and disgust. Moral emotions in environmental psychology are emotions based on ecological norms or ecological responsibility.

First, there are few studies on the relationship between EV and MA, and we refer to the related studies on protective values and MA. Protective values refer to the concept of refusing to exchange economic values. He and Xi (2005) found that in Chinese culture, protective values include “protective values about the natural environment and traditional cultural values” (such as destroying forests, polluting rivers and destroying monuments) and “protective values related to human relations, human rights and interpersonal emotions” (such as not caring for the elderly, hurting children, or cloning technology); the former are more valued and universal than the latter (He and Guan, 2005). Recent studies have found that monetary compensation is used to force people to exchange these protective values or make them “pay” for something important (participants were asked how much they can accept the disappearance of some species), which can lead to anger and disgust among individuals in response to this kind of exchange (Atran and Ginges, 2012; Sandel, 2012; Duc et al., 2013). According to the sacred value protection model (SVPM; Tetlock, 2003), because protective values are closely related to the self and moral identity, when individuals are forced to choose between values and monetary interests, they may be regarded as a threat to the self and moral identity. This exchange will aggravate negative emotions (Yue et al., 2021). Environmental economics also holds that “sacrificing natural or rare species for money” is morally wrong. Based on the above studies, we believe that MA will arise when an individual’s EV is threatened (such as through the destruction of the natural environment, the extinction of marine species, and the emission of nuclear wastewater into the ocean).

Second, the importance of moral emotions for ecofriendly decision-making and behavior has been proven (Harth et al., 2013). For example, Melissa and Janet (2020) found that ecoguilt can motivate environmental behavior change. Despite the established relationship between guilt and PEB across diverse domains, other-oriented emotions have been infrequently used as predictors of individual environmental behaviors, and in particular, MA is rare. A qualitative study found students to be very concerned about nature, to express empathy for the harm suffered by nature, and to be angry with humans who destroy the environment (Herman et al., 2018). According to the model of environmental justice, anger over violating ecological norms affects people’s willingness to adopt environmentally friendly behaviors. Tapia-Fonllem et al. (2013) measured residents’ anger toward other people’s bad environmental behaviors (such as felling trees, littering, or wasting water resources) and found that anger has a significant impact on PEB (including green purchases, energy and water savings, recycling of goods, and participation in environmental protection activities). Nerb and Spada (2001) asked participants to report their levels of anger and sadness after reading news that the environment was being damaged by human activities and found that only the emotion of anger had significant predictive power for PEB (for instance, donations for repairing the environment).

Based on previous studies, we propose the following hypotheses:

H4. **Individuals’** EV **is positively related to** MA.

H5. **Individuals’** MA **is related to** PEB.

### The Relationship Between Risk Perception and Moral Anger

Regarding the emotional reaction to risk perception, Böhm (2003) divides emotions into other- and self-related emotions, both of which are based on the perception of environmental risks and ethics. The former refers to expressing anger toward others and attributing responsibility to others who destroy the environment. The latter refers to personal guilt or shame, meaning that individuals blame themselves rather than others. The analysis results show that an individual’s responsibility for environmental risks has a positive impact on self-related moral emotions, including guilt and shame, instead of other-related emotions (Adams et al., 2020; Jeong et al., 2021). However, some scholars present different views. According to the social-cognitive theory model, the cognitive factors of risk (probability of event occurrence, event severity, or efficiency of event processing) trigger emotional reactions (anxiety, panic, anger). When responsibility for risk can be clearly assigned to a third party, this will produce ethical other-oriented emotions (such as anger). For example, discharging nuclear wastewater into the ocean will arouse people’s anger. As Ojala (2007) said, when individuals understand global environmental threats, this may trigger unpleasant emotions.

**Based on the above, we assume the following:**

H6. **Individuals’** RP **is related to** MA.

### Study Hypotheses

**FIGURE 1:**
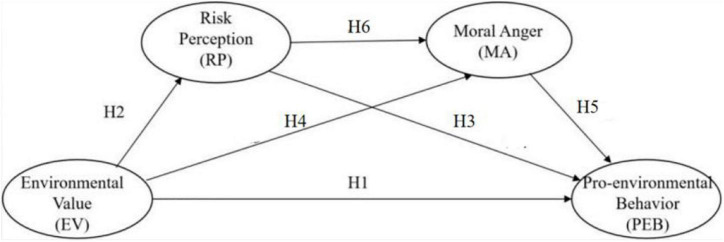
Conceptual model of hypothesis path (H) indicating causal relationships between each variable.

In this study, a chain mediation model (Figure 1) was applied to test the mediating role of RP and MA in the association between EV and PEB. Specifically, three mediation paths were examined as follows:

**Hypothesis 7: Environmental value is indirectly associated with young adults’** PEB ***via*** RP.

**Hypothesis 8: Environmental value is indirectly associated with young adults’** PEB ***via*** MA.

**Hypothesis 9: Environmental value is indirectly associated with young adults’** PEB **through** RP **and** MA.

## Materials and Methods

### Participants

A total of 600 college students from four universities in Beijing, Inner Mongolia, Yunnan, Fujian Province, China were selected using the convenience sampling method. Survey data were obtained through written network measurement. Voluntary participation and anonymity were emphasized. To ensure the objectivity and reliability of the data obtained, we promised that the data obtained from the questionnaire would only be used for academic research and that the participants could fill out the survey according to their actual situations. There were no right or wrong answers. The survey was conducted in October 2020. Data were cleaned to omit people who completed the survey too quickly (*n* = 19) or provided incomplete responses (*n* = 13).

A total of 568 college students returned completed questionnaires, representing an effective response rate of 94.6%. Of the final sample, 305 (55%) were male, 253 (45%) were female, 94 (16.8%) were urban residents, 464 (83.2%) were rural residents, 477 (86%) were Han of descent, 81 (14%) were ethnic minorities, 350 (59.8%) majored in art, and 235 (40.2%) majored in science. The mean age of the participants was 19.59 years (*SD* = 1.28). The proportions of participants in years 1–4 in university were 53.5, 23.5, 12.9, and 10.1%, respectively.

### Measures

#### Environmental Value Scale

Four items (Steg et al., 2014) were used to measure EV and have been used extensively in previous research on public attitudes toward climate change and PEB (see, e.g., Poortinga et al., 2004; Slimak and Dietz, 2010). Participants rated the importance of the following four items: preventing pollution (protecting natural resources); respecting the earth (harmony with other species); unity with nature (fitting into nature); and protecting the environment (preserving nature). The scale ranged from −1 “not important” to 4 “of supreme importance.” The higher the score was, the higher the degree of EV was, and the greater the inclination to protect the environment was. The scale showed good internal consistency (Cronbach’s α = 0.93), so a single score measuring pro-EV was computed and used for subsequent analyses. The validity index of the questionnaire reached an acceptable level (χ^2^/*df* = 3.75, CFI = 0.99, TLI = 0.99, RMSEA = 0.07, RMR = 0.007).

#### Risk Perception Scale

Risk perception was assessed using six items adopted from the environmental RP scale (Zhang, 2013) on a five-point Likert-type scale. This ten-item measure measures three dimensions: (1) perception of severity (e.g., “The destruction of the natural environment will have a serious impact on my life”) refers to the perceived seriousness of the impact of environmental hazards on human society; (2) perception of durability (e.g., “Once the natural environment is destroyed, it will last for a long time”) refers to the perceived persistence of environmental hazards; and (3) perception of possibility (e.g., “There is a great possibility that the natural environment will deteriorate”) refers to the possibility of environmental hazards. The higher a score is, the stronger perceptions of environmental threat are. For the current sample, Cronbach’s alpha for the entire questionnaire was measured as 0.85. The validity fitting indexes of the questionnaire were measured as χ^2^/*df* = 4.44, CFI = 0.95, TLI = 0.95, RMSEA = 0.079, and RMR = 0.027.

#### Moral Anger Scale

To measure the MA of the participants, a 4-item scale was modified from the work of Beugré (2012). Example items include the following: I feel sad, angry, bothered, and concerned when I see others destroying the environment. The items were measured on a 5-point Likert scale ranging from “1-strongly disagree” to “5-strongly agree.” For the current sample, Cronbach’s alpha for the entire questionnaire was measured as 0.95. The validity fitting indexes of the questionnaire were measured as χ^2^/*df* = 4.41, CFI = 0.99, TLI = 0.99, RMSEA = 0.078, and RMR = 0.011.

#### Pro-environmental Behavior Scale

Pro-environmental behavior was assessed using five items adapted from the research of Liu and Wu (2013) on a five-point frequency scale ranging from “1-never” to “5-always.” The present study only uses items for private domain PEB, which are closely related to the actual lives of university students with strong practicality and high content validity and which are used as the measurement indicators of university students’ PEB in this study. For the current sample, internal consistency was measured as 0.82. The validity fitting indexes of the questionnaire were measured as χ^2^/*df* = 3.72, CFI = 0.99, TLI = 0.97, and RMSEA = 0.08.

#### Demographic Characteristics

Because previous research has indicated that a person’s gender, urban or rural residence, college major, and education level may affect human PEB (Zhang et al., 2019), we included these four variables as control variables.

#### Analytical Strategies

In this analysis, we validated the hypothetical model using a three-step strategy. First, the reliability and consistency of the questionnaire data were tested. Second, the measurement model was validated by confirmatory factor analysis (CFA), and third, the fitting and path coefficients of the hypothetical model were measured by structural equation modeling. We used SPSS 17.0 and Mplus 7.4 for our SEM analysis.

The reliability and consistency of the questionnaire data were tested before conducting the SEM analysis. We used Cronbach’s α coefficients to measure reliability. According to the results, all Cronbach’s alpha (a) coefficients were between 0.82 and 0.95, exceeding the standard value of 0.7 and indicating that the scale was reliable.

Confirmatory factor analysis is part of the structural equation model. Thompson (2007) proposed that CFA should be completed to test for structural validity before analyzing the structural equation model. Construct validity includes convergence validity and discrimination validity. The results show that the factor loadings ranged between 0.6 and 0.9. The combined reliability (CR) was between 0.96 and 0.97 and exceeded the standard value of 0.7, and all average variance extracted (AVE) values were between 0.70 and 0.98 and exceeded the standard value of 0.5. Therefore, the questionnaire exhibits appropriate convergent and discriminant validity.

## Results

### Common Method Bias Test

Since multiple variable data used in this study were from the same participant, there may be a common method bias problem. In this study, single method-factor approaches were used for a common method bias test (Xiong et al., 2012). This method extracts the “general” factor and then incorporates it into the structural equation model. The results indicate that the fitting of the model is unsatisfactory at χ^2^/*df* = 5.20, CFI = 0.80, TLI = 0.80, and RMSEA = 0.09. To a certain extent, this shows that common method **bias** is not extreme for this study.

### Descriptive and Pearson Correlation Results

The descriptive statistics and Pearson correlations for the assessed variables are presented in Table 1. All variables were found to be significantly correlated.

**TABLE 1:** Descriptive statistics and Pearson correlations between the study variables.

	*M* ± *SD*	1	2	3	4	5	6	7
1. EV	4.27 ± 0.75	1						
2. POS	3.70 ± 0.55	0.349**	1					
3. POD	4.24 ± 0.71	0.426**	0.729**	1				
4. POP	4.21 ± 0.74	0.375**	0.662**	0.734**	1			
5. RP	4.05 ± 0.59	0.430**	0.863**	0.922**	0.904**	1		
6. MA	4.18 ± 0.75	0.499**	0.535**	0.567**	0.487**	0.588**	1	
7. PEB	3.58 ± 0.80	0.347**	0.393**	0.321**	0.268**	0.356**	0.449**	1

**p < 0.01. EV, environmental value; RP, risk perception; POS, perception of severity; POD, perception of durability; POP, perception of possibility; MA, moral anger; PEB, pro-environmental behavior.

### Model Fit Tests

When a structural equation model is used to validate theoretical models, a satisfactory model fit is a necessary condition (Byrne, 2001). This study adopts the values of χ^2^/df, CFI, GFI, TLI, IFI and RMSEA to estimate model fit. All of these indices exceed the standard value (χ^2^/*df* = 4.16, CFI = 0.99, GFI = 0.98, TLI = 0.97, IFI = 0.99, and RMSEA = 0.075). Consequently, we can conclude that the model we propose has a good fit with the data.

### Structural Results

**FIGURE 2:**
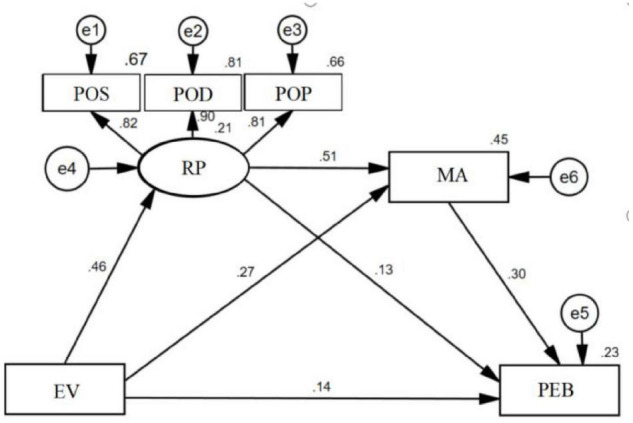
Hypothesis test results.

The structural results are presented in Figure 2. The results support our six hypotheses. The results for the positive and direct effects of EV on PEB (standardized direct effect β = 0.14, *t* = 3.119, *p* < 0.05) are statistically significant, and the 95% confidence interval is [0.270, 0.420], supporting **Hypothesis 1**. The results for the positive and direct effects of EV on RP (standardized direct effect β = 0.46, *t* = 10.781, *p* < 0.05) are statistically significant, and the 95% confidence interval is [0.389, 0.560], supporting **Hypothesis 2**. RP has significant positive impacts on PEB (standardized direct effect β = 0.13, *t* = 2.401, *p* < 0.01), and the confidence interval of the path is [0.163, 0.361], supporting **Hypothesis 3**. The results indicate that EV can positively predict MA (standardized direct effect β = 0.27, *t* = 7.191, *p* < 0.01), and its 95% confidence interval is [0.436, 0.584], supporting **Hypothesis 4**. MA has significant positive impacts on PEB (standardized direct effect β = 0.30, *t* = 5.770, *p* < 0.05), and the confidence interval of the path is [0.206, 0.414], supporting **Hypothesis 5.** The results indicate that RP can positively predict MA (standardized direct effect β = 0.51, *t* = 12.114, *p* < 0.01β = 0.51, *p* < 0.05), and its 95% confidence interval is [0.410, 0.572], supporting **Hypothesis 6**.

As shown in Figure 2, there are not only direct effects between EV and PEB but also indirect effects between them. Three mediating effects may be at play in the research model. First, RP may act as an intermediary between EV and PEB. Second, MA may act as an intermediary between EV and PEB. Third, RP and MA may play multiple intermediary roles in the relationship between EV and PEB. Therefore, we further analyze these possible mediating effects.

### Mediating Effect Analysis

This study controls demographic variables gender, age, birthplace, and academic major and uses a structural equation model to verify the mediating roles of environmental RP and MA in EV and proecological behaviors. We used bootstrapping (Wen and Ye, 2014) to test the mediating variable effects of RP and MA. We performed bootstrapping at a 95% confidence interval with 2,000 samples (Taylor et al., 2008). We calculated the asymptotic critical ratio (t) and confidence interval of the lower and upper bounds (95% BC, 95% percentile) to test whether the indirect effects were significant (Preacher and Hayes, 2008). There is an indirect effect when *t* > 0, and the 95% confidence interval does not contain zero. First, we test whether the total indirect effect is significant.

The confidence interval of the chain mediation effect from EV to PEB is [0.045, 0.107]. This interval does not contain zero, which shows that the chain mediation effect is significant. The three mediation paths in the model are such that EV is indirectly associated with young adults’ PEB *via* RP (Hypothesis 7), EV is indirectly associated with young adults’ PEB *via* MA (Hypothesis 8), and EV is indirectly associated with young adults’ PEB through RP and MA (Hypothesis 9). The results of the significance test of the three mediation paths are shown in Table 2. Therefore, the total effects of EV on PEB include 17.03% of the mediating effect through RP, 23.06% of the mediating effect through MA, and 20.04% of the mediating effect through RP and MA.

**TABLE 2:** Direct, indirect and total effects†of the SEM components depicted in Figure 2.

Effect source	Standardized path effect	Percentage effect	BC 95%	
			Lower	Upper
Indirect effects	0.21118	60.13%	0.160	0.260
EV—RP—PEB	0.46 × 0.13 = 0.0598	17.03%	0.016	0.129
EV—MA—PEB	0.27 × 0.30 = 0.081	23.06%	0.039	0.110
EV—RP—MA—PEB	0.46 × 0.51 × 0.30 = 0.07038	20.04%	0.045	0.107
Direct effects	0.140	39.87%	0.042	0.228
Total effects	0.35118	100%	0.270	0.420

## Discussion

One of the primary findings of this research is that EV is related to PEB. This result is in line with the argument made by Ünal et al. (2018) that people with higher EV are more likely to behave in an environmentally friendly manner.

As most countries face great pressures to improve the environment, this study attempts to better understand ways to transform individual EV into PEB. Many studies on PEB come from Western countries (Casaló and Escario, 2018; Park, 2019). In recent years, research on environmental behavior among Chinese people has begun in China (Wang and Wu, 2015; Zhou et al., 2018; Fang, 2020).

This study explores college students’ PEB in the Chinese context. The results confirm the relationships between EV, RP, MA, and PEB found in previous studies. First, the study shows that EV can directly predict PEB. This finding is consistent with other study results (Riper and Kyle, 2014; Pradhananga et al., 2017; Sheng et al., 2020); individuals with high EV are more likely to engage in PEB. Since ancient times, Chinese people have advocated the ecological view of the unity of humans and nature, the equality of all living beings, and conformity to nature. This ecological view also profoundly guides (restricts) the environmental behavior of college students. The results also show that EV is positively correlated with RP and MA, which is consistent with past results (Stoutenborough et al., 2014; Ojala and Lidskog, 2017). This means that college students who believe it is important to protect the environment are better able to perceive environmental threats and are more morally angry with others for violating ecological norms (littering, mistreating small animals, or eating wild animals). Frantz and Mayer (2010) also supports this view and find that altruistic people (i.e., those concerned about the interests of others and of other species) are more attuned to global environmental risks (such as ozone holes and global warming). Second, we found a significant positive correlation between RP and PEB, which is consistent with the existing research results (Joireman et al., 2004; Xie et al., 2019). The stronger the perception of environmental risk is, the more likely an individual is to engage in PEB. For example, when individuals have a strong sense of threat to the environment, they are more willing to change their purchase decisions and behavior for environmental reasons, and they are more likely to buy green products (Wang and Li, 2019). People more attuned to risks of urban smog will realize that smog causes respiratory diseases and are more likely to take actions associated with reducing smog (Zhu et al., 2020). Third, our results show a positive correlation between RP and MA where individuals with greater RP tend to think that human beings do too much harm to the natural environment and show strong levels of MA. In addition, MA will encourage individuals to protect the environment. In a model study of responsible ecological behavior, MA was found to be the most potent predictor of PEB (Montada and Kals, 2000).

Further analysis is needed to determine the role of RP and MA in EV and PEB. The results show that RP plays a mediating role between EV and PEB. Its chain mode is EV—RP—PEB. EV is indirectly associated with young adults’ PEB *via* RP, which is consistent with the results of previous studies (Nordlund and Garvill, 2002; Zhang, 2013; Jeong et al., 2021). This means that individuals who attach importance to EV are more likely to perceive threats from the environment and pay more attention to environmental issues, which can easily transform into environmental behaviors to guard against risks. Therefore, environmental RP is more related to whether individual EV can be transformed into natural environmental behaviors, reflecting the human psychological representation of environmental hazards.

Another important finding of our study is another meditating chain between EV and PEB: EV—MA—PEB. EV is indirectly associated with young adults’ PEB *via* MA. Individuals with high EV will experience more MA when they see others destroy the environment, and this moral emotion encourages individuals to engage in more PEB. Individuals with high EV will experience more MA when they see others destroy the environment, and this moral emotion will prompt individuals to engage in more PEB. In addition, the results show that the mediating effect of MA is greater than that of the other two paths. This means that emotional factors play an essential role in transforming EV into PEB, and emotional aspects can better explain the relationship between EV and PEB than cognitive factors (Kanchanapibul et al., 2014; Wang and Wu, 2015). Liu et al. (2020) also believed that the influence of the emotional system on environmental behavior is dominant. In environmental decision-making, individuals do not necessarily act in a rational way but may also be guided by emotional and moral principles (Onwezen, 2015; Han et al., 2018).

The fourth important finding of this study is that RP and MA play a significant chain mediating role between EV and PEB. The path is EV—RP—MA—PEB. In other words, individuals with high EV have a stronger ability to perceive environmental threats and then experience more MA against those who cause environmental risks, prompting them to engage in PEB. Onwezen et al. (2013) and Han et al. (2018) argue cognitive and emotional factors are the core mediating variables between EV and PEB. To some extent, emotion is the result of the cognitive process. This is consistent with existing studies that emphasize the critical role of cognitive-emotional relationships in individual decision-making processes, not only for consumer behavior but also for environmental behavior (Hunter, 2006; Bamberg et al., 2007; Wang and Wu, 2015). Our results support this conclusion and further find that psychological factors, including people’s cognition and moral emotion toward environmental problems, are positively related to their environmental behavior intention. RP and MA work together to some extent, motivating us to engage in behavior that is good for the environment.

Although cognitive and emotional factors play an essential role in explaining the formation of individual environmental behavior, few empirical studies combine the two to understand the mechanism of environmental behavior. This study brings cognitive and emotional factors into a comprehensive theoretical framework, which greatly deepens our understanding of PEB. In other words, this present study makes effective use of the driving forces of PEB. It successfully examines the complex relationship between these variables to better understand the mediating effects of EV and young adults’ PEB. It provides not only a reference for Chinese environmental psychology but also data support for exploring related theoretical mechanisms such as VBN theory and PEB theory.

The practical significance of this study is as follows. First, societies and schools can cultivate the ecological values of college students and the public through environmental education, raise people’s awareness of environmental problems, and motivate people pay more attention to the interests of the ecosystem and the wellbeing of nature. Second, we suggest that the media provide more news about ecological crises and environmental issues (the threats of urban haze and shortages of resources). Such efforts can help raise awareness of environmental risks and encourage people to take action to protect the environment. Third, many public service advertisements use knowledge of EV and RP to promote environmental protection behavior when designing and promoting themes. For example, in global warming advertisements, the earth is portrayed as warming; similar to ice cream, it is constantly melting, and finally, delicate ice cream, like the earth, disappears. Advertisements can emphasize the destruction of the human environment and the threat of environmental damage to our survival. It is crucial for people to support EV. By paying attention to the interests of these biospheres, we can stimulate EV, individual awareness of risks, and MA at violations of ecological norms, which will eventually enhance pro-environmental intentions and behavior.

## Conclusion

The main objectives of this study were to examine EV, RP, and MA in relation to PEB. Consistent with the literature, our research confirms that EV positively relates to PEB. Furthermore, our research empirically demonstrates that individuals with higher EV have higher levels of RP, more moral emotions, and more PEB. Based on these results, we recommend that societies and families cultivate university students’ EV. Practitioners in the field of the environment need to consider providing the public with scientific knowledge of risks related to environmental issues to promote environmental protection. The novel finding of the current study is that RP and MA play a significantly chaining mediating role in the relationship between EV and PEB. These results imply that it is necessary to understand human behavioral psychology at the psychological level and that a scientific approach is needed to activate the PEB of college students to maintain environmental sustainability.

The results of this study show that the mediating effects of RP and MA may contribute to understanding the relationship between EV and PEB in a sample of Chinese individuals. However, this study has the following limitations that need to be addressed in future research. First, we use cross-sectional data, which may have endogeneity issues, which is a common problem in empirical research based on single survey data. Therefore, longitudinal data or causal experiments should be used in future studies to validate the claims of the present study. Second, we studied a sample of Chinese college students, excluding other groups. The results of this study could be verified in other populations in future studies. Third, part of the theoretical model described in this study is based on values-belief-norm theory. However, due to the limitations of the data obtained, we did not consider norms in the model for our analysis. Although our research focuses on the relationships between EV, RP, MA, and PEB, we can integrate environmental knowledge and norms into a complete VBN model for further research in the future.

## Data Availability Statement

The original contributions presented in the study are included in the article/supplementary material, further inquiries can be directed to the corresponding author/s.

## Ethics Statement

The studies involving human participants were reviewed and approved by the Inner Mongolia Normal University. The patients/participants provided their written informed consent to participate in this study.

## Author Contributions

XL contributed to the experimental design and analysis of the data and drafted the manuscript. ZL helped revise manuscript. TW provided final approval of the manuscript. All authors contributed to the article and approved the submitted version.

## Conflict of Interest

The authors declare that the research was conducted in the absence of any commercial or financial relationships that could be construed as a potential conflict of interest.

## Publisher’s Note

All claims expressed in this article are solely those of the authors and do not necessarily represent those of their affiliated organizations, or those of the publisher, the editors and the reviewers. Any product that may be evaluated in this article, or claim that may be made by its manufacturer, is not guaranteed or endorsed by the publisher.
